# Standardized Self-Report Tools in Geriatric Medicine Practice: A Quality Improvement Study

**DOI:** 10.1093/geroni/igab046.2397

**Published:** 2021-12-17

**Authors:** Melissa Northwood, George Heckman, Nicole Didyk, Sophie Hogeveen, Amanda Nova

**Affiliations:** 1 McMaster University, Hamilton, Ontario, Canada; 2 University of Waterloo, Waterloo, Ontario, Canada

## Abstract

Comprehensive geriatric assessment (CGA)—a multidimensional diagnostic process to determine medical, cognitive, and functional capacity—has historically included a narrative history supplemented by use of tools to assess domains such as mood or cognition based on assessor preference. This approach to CGA likely works to assess individuals but with increasing clinical complexity and frailty among older adults, a non-standardized approach may mean that key issues are not assessed, and program quality cannot be determined. The COVID-19 pandemic added to these challenges as social distancing practices meant limited face-to-face appointments and use of phone and video assessments. This quality improvement study implemented the interRAI Check-Up Self-Report instrument through a software platform in a specialized geriatric services practice. The instrument can be used over the phone and summarizes specific health problems and needs as well as information about caregiver status and financial trade-offs. Focus groups were also conducted with specialized geriatric services interprofessional team to explore their experiences with implementation. The descriptive analysis of the self-report data revealed expected geriatric issues, such as cognitive and functional impairment, falls and pain. Clients were also commonly experiencing medical instability, cardiorespiratory symptoms, communication impairments, and elevated risk for emergency department visit. Staff found the self-report tool feasible, easy to use, efficient, and the program-level metrics helpful for program planning. In conclusion, introduction of a standardized self-report enhanced CGA by creating a systematic method to flag, track, and prioritize all areas of need for immediate and future care planning at both the client and program level.

